# Discriminable spatial patterns of activation for faces and bodies in the fusiform gyrus

**DOI:** 10.3389/fnhum.2014.00632

**Published:** 2014-08-14

**Authors:** Na Yeon Kim, Su Mei Lee, Margret C. Erlendsdottir, Gregory McCarthy

**Affiliations:** Human Neuroscience Laboratory, Department of Psychology, Yale UniversityNew Haven, CT, USA

**Keywords:** face area, body area, fusiform gyrus, multivoxel pattern analysis, fMRI

## Abstract

Functional neuroimaging studies consistently report that the visual perception of faces and bodies strongly activates regions within ventral occipitotemporal cortex (VOTC) and, in particular, within the mid-lateral fusiform gyrus. One unresolved issue is the degree to which faces and bodies activate discrete or overlapping cortical regions within this region. Here, we examined VOTC activity to faces and bodies at high spatial resolution, using univariate and multivariate analysis approaches sensitive to differences in both the strength and spatial pattern of activation. Faces and bodies evoked substantially overlapping activations in the fusiform gyrus when each was compared to the control category of houses. No discrete regions of activation for faces and bodies in the fusiform gyrus survived a direct statistical comparison using standard univariate statistics. However, multi-voxel pattern analysis differentiated faces and bodies in regions where univariate analysis found no significant difference in the strength of activation. Using a whole-brain multivariate searchlight approach, we also found that extensive regions in VOTC beyond those defined as fusiform face and body areas using standard criteria where the spatial pattern of activation discriminated faces and bodies. These findings provide insights into the spatial distribution of face- and body-specific activations in VOTC and the identification of functionally specialized regions.

## INTRODUCTION

The ability to extract biologically relevant information from faces and bodies is critical for social interactions among humans and for many nonhuman animals. Single-cell recording in sheep and in monkeys has revealed that some temporal lobe neurons respond selectively to faces (Gross et al., 1972; Perrett et al., 1982; Kendrick and Baldwin, 1987; Tanaka et al., 1991), hands (Desimone and Albright, 1984), or headless bodies (Wachsmuth et al., 1994). In humans, there is converging evidence that faces activate regions of ventral occipitotemporal cortex (VOTC), and in particular a region of the lateral mid-fusiform gyrus (e.g., Sergent et al., 1992; Allison et al., 1994; Haxby et al., 1994; Puce et al., 1995; McCarthy et al., 1997). This latter region has been shown to respond selectively to faces when compared to a variety of non-corporeal control stimuli, such as scenes, objects, letter strings, and textures. Indeed, such apparent selectivity has led to its widely adopted functional designation as the fusiform face area, or FFA (Kanwisher et al., 1997).

Areas selective to bodies have also been reported in studies using fMRI. Downing et al. (2001) reported a region of lateral occipitotemporal cortex (LOTC) to be selectively activated by bodies without faces (a region they designated as the extrastriate body area, or EBA). In a later study, Peelen and Downing (2005) reported a similar body-selective area along the VOTC in the fusiform gyrus that they designated as the fusiform body area, or FBA. The selectivity for bodies in the FBA has been studied by comparing the response to bodies or body parts to non-corporeal objects (Taylor et al., 2007; Hodzic et al., 2009; Vocks et al., 2010; Willems et al., 2010; Ewbank et al., 2011), object parts (Costantini et al., 2011), or scrambled bodies (Aleong and Paus, 2010).

Evidence for anatomically distinct FFA and FBA would be consistent with a modular neural organization, as has been previously proposed for face processing (Kanwisher et al., 1997). However, if the same voxels respond equally to faces and bodies, a more distributed organization may be considered. Studies in the macaque using fMRI have found multiple clusters in the superior temporal sulcus (STS) of the macaque brain that respond to faces (Tsao et al., 2003), and an adjacent and overlapping region that responds to body parts (Pinsk et al., 2005, 2009). In fMRI studies that have compared activations in human VOTC evoked by faces and bodies, evidence for the anatomical distinction of these areas has been equivocal. For example, Spiridon et al. (2006) reported that the activation evoked by body stimuli was not statistically significantly different than that evoked by faces in the FFA. Morris et al. (2008) found no statistical difference in a VOTC region corresponding to the FFA when contrasting activations evoked during guided eye fixations of the face or body of a static image of a male human avatar. The time courses of activity confirmed that faces and torsos evoked no differential activation in this region, but hands evoked much less activation. This finding was consistent with an earlier report by Morris et al. (2006), which found that bodies with naturally occluded faces and faces with naturally occluded bodies equally activated a lateral region of the VOTC corresponding to the FFA. However, in both studies by Morris et al. (2006, 2008), viewing bodies with occluded faces or making guided fixations upon a torso activated a region adjacent and medial to the FFA. Morris et al. (2006) note that the medial VOTC areas activated by bodies without faces were the same as those previously identified with differential processing of objects and textures, suggesting that these activations might represent domain-general processes.

In a recent intracranial event-related potential study, recordings from subdural electrodes along the VOTC including the fusiform gyrus were compared for faces, bodies, and eyes (Engell and McCarthy, 2014). While many sites in this region showed strong selectivity to these corporeal stimuli compared to a control category, most sites that responded to one of these three stimulus categories responded to the other two. However, the authors also showed shifts in the spatial distribution of voltage associated with faces, bodies and eyes at adjacent electrode sites, suggesting that a different configuration of current sinks and sources is engaged by these stimulus types. This finding suggests a differential neural organization among the three categories at a finer spatial scale.

Others, however, have made a stronger argument in favor of separate selective face and body areas, while also noting regions of overlap. Using higher spatial resolution than most contemporaneous studies, Schwarzlose et al. (2005) initially defined the FFA on the basis of the face > object contrast, and the FBA on the basis of the body > object contrast. They then defined face- or body-selective regions by eliminating overlapping voxels that were included in both the FFA and FBA from the initial contrasts. The time courses of activation in these non-overlapping areas demonstrated the regions’ selective response to either faces or bodies. Weiner and Grill-Spector (2010) reported minimally overlapping, but rather alternating face and body activations within VOTC instead of a single specialized area for faces or bodies. However, while both studies reported the region’s response to faces compared to objects and bodies compared to objects, they did not directly contrast faces and bodies to each other. Indeed, activations evoked by faces and bodies have rarely been statistically compared to each other when those regions are identified. Thus it remains unclear whether the areas defined as selective had significantly different levels of activation.

The studies reviewed thus far have focused on identifying discrete selective regions that respond only to faces, or only to bodies, and have thus deemphasized the regions where the activations for faces and bodies overlap. An alternative perspective is that faces and bodies may be represented in activation *patterns* within a larger area of VOTC, rather than in discrete regions such as the FFA or FBA (see Haxby et al., 2001, for evidence supporting a pattern perspective for face processing). Multi-voxel pattern analysis (MVPA) has been employed to determine whether sufficient information exists within local brain regions to classify a stimulus into one of a number of different categories (e.g., Haxby et al., 2001; Connolly et al., 2012), and to investigate the functional organization of the regions at a finer scale (Downing et al., 2007). Peelen and Downing (2007) have suggested that MVPA reveals more subtle functional differences in activations that overlap at a larger spatial scale.

Here, we examined the activation to faces and bodies at high spatial resolution in a sample of 21 young adults. Our focus was upon the fusiform gyrus and adjacent VOTC regions, with the goal of determining the degree of overlap between face and body activations, and the degree to which faces and bodies can be discriminated within regions of overlap. Using a univariate general linear model (GLM) approach, we first tested whether discrete regions of the fusiform gyrus were activated when faces and bodies were statistically compared. We then used MVPA to determine whether sufficient information was present in the pattern of activation in areas where both faces and bodies evoked overlapping and statistically indistinct activation to classify a stimulus as a face or body. Finally, we conducted a whole-brain multivariate searchlight analysis to identify all regions in the brain where faces and bodies could be discriminated.

## MATERIALS AND METHODS

### SUBJECTS

Twenty-one healthy adults (13 female, mean age 23.7 ± 4.0 years, all right-handed) with normal or corrected-to-normal vision and no history of neurological or psychiatric illnesses participated in this study. All participants gave written informed consent. The Yale Human Investigations Committee approved the protocol.

### EXPERIMENTAL DESIGN

**FIGURE 1:**
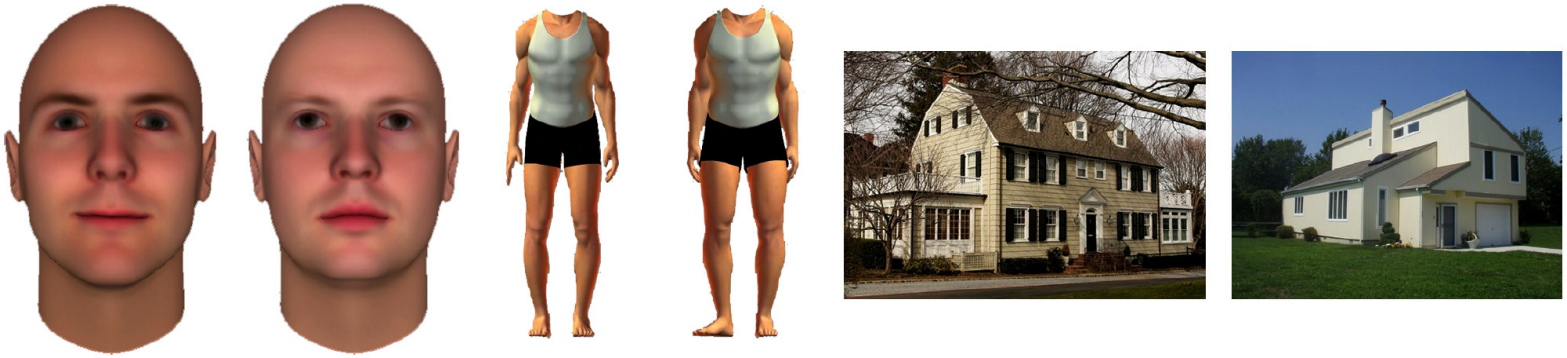
**Example images of faces, bodies, and houses.** Stimuli were presented in 12-s blocks in pseudo-randomized order. Each run consisted of 12 stimulus blocks (four blocks for each stimulus category), and each participant completed four runs. Participants were instructed to count the number of times they saw the same picture twice consecutively. No button press was required.

**Figure 1** presents exemplars of the stimuli used in the experiment. Face stimuli were created using FaceGen software (Singular Inversions, Toronto, ON, Canada). Body stimuli were created using Poser 6.0 (Curious Labs Inc., Santa Cruz, CA, USA). House stimuli were photographs of houses with natural scenes in the background. All stimuli were presented on the center of a screen (10° × 10°) located behind the participant in the scanner and viewed with a mirror mounted in the head coil.

Each participant completed four runs, each of which lasted 4 min 54 s. Each run consisted of a pseudo-randomized block design in which 12-s stimulus blocks were interleaved with 12-s blocks of fixation. A total of 12 stimulus blocks were presented in every run, including four each for faces, bodies, and houses. Stimulus blocks consisted of eight images from a single category. Stimuli were presented for 1 s each, interleaved with 500 ms of fixation. Participants were instructed to count the number of times they saw the same picture twice consecutively. No button press was required.

### fMRI IMAGE ACQUISITION AND PREPROCESSING

Data were acquired at the Magnetic Resonance Research Center at Yale University using a 3.0 T Siemens TIM Trio scanner with a 32-channel head coil. Functional images were acquired using a multiband imaging sequence (TR = 2000 ms, TE = 32 ms, flip angle = 62 , FOV = 210 × 202 mm, matrix = 104 × 100, slice thickness = 2.0 mm, 60 slices, multiband accelerate factor = 3) yielding isotropic voxels that were 2 mm^3^. Two structural images were acquired for registration: T1 coplanar images were acquired using a T1 Flash sequence (TR = 335 ms, TE = 2.61 ms, flip angle = 70°, FOV = 240 mm, matrix = 192 × 192, slice thickness = 2.0 mm, 60 slices), and high-resolution images were acquired using a 3D MP-RAGE sequence (TR = 2530 ms, TE = 2.77 ms, flip angle = 7°, FOV = 256 mm, matrix = 256 × 256, slice thickness = 1 mm, 176 slices).

### ANALYSIS OVERVIEW

The data were analyzed using several different methods so that our observations could be compared to previously reported findings. We first conducted a conventional univariate GLM to obtain parameter estimates for each condition and whole-brain statistical maps for the contrasts of interest: face > house, body > house, face > body, and body > face. We examined the face > house and body > house activation maps and measured the overlap between the two. Because our focus in this paper is upon the VOTC, we used the temporal occipital fusiform cortex (TOFC) overlay from the Harvard–Oxford Structural Atlas as an anatomical mask, or region of interest (ROI), for several of our analyses. Each hemisphere was analyzed separately.

Using a beta series derived from hemodynamic model fitting, we used MVPA to test whether activation patterns in the overlapping region could discriminate faces, bodies, and houses. We also compared each subject’s uncorrected contrast maps in the subject’s own anatomical space, as some previous studies reported face- and body-specific activations on a subject-by-subject basis (e.g., Schwarzlose et al., 2005; Weiner and Grill-Spector, 2010).

Finally, we conducted a multivariate searchlight analysis of the whole brain to discover regions that could discriminate faces from bodies that fall outside of the fusiform regions that were the focus of our initial analyses.

#### Image preprocessing

Image preprocessing was performed using the FMRIB Software Library (FSL; http://www.fmrib.ox.ac.uk/fsl). Structural and functional images were skull-stripped using the Brain Extraction Tool (BET). The first three volumes (6 s) of each functional dataset were discarded to allow for MR equilibration. Functional images then underwent motion correction (using the MCFLIRT linear realignment) and high-pass filtering with a 0.01 Hz cut-off to remove low-frequency drift. Data were not spatially smoothed. The functional data were registered to the coplanar images, which were in turn registered to the high-resolution structural images, using non-linear registration, and then normalized to the Montreal Neurological Institute’s template (MNI152). For subject-specific analyses, each participant’s functional images were registered to the participant’s own high-resolution structural images.

#### Whole-brain contrast maps

Whole-brain voxel-wise GLM analyses were performed using FSL’s FMRI Expert Analysis Tool (FEAT). Each condition within each preprocessed run was modeled with a boxcar function convolved with a gamma hemodynamic response function. The model included explanatory variables (EVs) for the three stimulus types: faces, bodies, and houses, as well as confound EVs to exclude time points with excessive head motion (>2 mm) from analysis. Subject-level analyses combining multiple runs were conducted using a fixed effects model. Group-level analyses were performed using a mixed effects model, with the random effects component of variance estimated using FSL’s FLAME 1 + 2 procedure. Clusters were defined as contiguous sets of voxels with *Z* > 2.3 and then thresholded using Gaussian random field theory (cluster probability *p* < 0.05) to correct for multiple comparisons (Worsley et al., 1996). We also generated uncorrected statistical maps with *Z* > 1.96 for the subject-specific analyses as described below.

#### Subject-specific analysis

Whole-brain statistical maps were generated for each subject using an uncorrected threshold of *Z* > 1.96. Subject-specific ROIs were defined by transforming the Harvard-Oxford Atlas TOFC ROI into subject space. Within this ROI, we obtained the intersection of the face > house and body > house contrasts (i.e., the “overlap”) to identify voxels that respond to both faces and bodies. We also obtained the intersection of the face > house and face > body contrasts (“face specific”) to identify voxels that were selective to faces in both contrasts, and similarly, the intersection of the body > house and body > face contrasts (“body specific”). We then excluded “face specific” and “body specific” voxels from the “overlap” such that the remaining voxels (“exclusive overlap”) showed preferential response to both faces and bodies compared to houses, but did not respond differently between faces and bodies.

#### Multi-voxel pattern classification

MVPA was performed on the “exclusive overlap” (i.e., voxels in the overlap between the face > house and body > house contrasts that did not respond differently between faces and bodies) from the group-level uncorrected (*Z* > 1.96), unsmoothed statistical maps within the TOFC ROI.

To perform the pattern analysis, we first obtained parameter estimates (or betas) for each stimulus block and for each participant by using hemodynamic model fitting. Specifically, the preprocessed functional data were registered and normalized to the MNI 152 template using FSL’s Non-linear Image Registration Tool (FNIRT). Regression analyses were then performed using AFNI’s (Cox, 1996) 3dDeconvolve and 3dREMLfit functions, where each stimulus block was modeled using the BLOCK5 basis function with duration of 12 s. The resulting beta volumes for each stimulus block (16 beta volumes in total for each stimulus category) were concatenated into a single beta series for each subject.

A three-way classification (faces, bodies, and houses) was performed using a linear support vector machine (SVM) classifier, as implemented in PyMVPA (Hanke et al., 2009) on the beta series and only within the overlap ROI. Within each volume in the beta series, each voxel’s beta values were mean-normalized (by *Z*-scoring using the mean and standard deviation of the voxels within the overlap ROI), which effectively removed mean differences across volumes in the beta series. This was done to ensure that any MVPA differences found were based on spatial pattern differences and not mean activation level differences. Classification training and testing were performed using a leave-one-run-out cross-validation strategy. We ensured that each condition contained the same number of examples in the training and testing sets using PyMVPA’s Balancer. Confusion matrices were generated during classification to assess if the three-way classification discriminated all three categories successfully instead of only a subset of categories.

#### Whole-brain searchlight analysis

A whole-brain searchlight analysis was performed to identify all brain regions that discriminated between faces and bodies (Kriegeskorte et al., 2006). For each participant, voxels were extracted from a spherical searchlight with a two-voxel radius (33 voxels in each searchlight including the central voxel) and MVPA was performed. The searchlight then moved through each voxel in the brain. We examined pair-wise classification performance (faces vs. bodies) rather than three-way classification (faces vs. bodies vs. houses) because distinct patterns of activity evoked by houses led to higher classification performance in regions medial to the fusiform gyrus. As in ROI-based multivariate analyses described above, the data were normalized to remove mean activation differences between categories. A linear SVM classifier was trained and tested using the data from each searchlight, using a leave-one-run-out cross-validation strategy. The classification accuracy of each searchlight was assigned to the central voxel in the sphere, yielding an image of whole-brain classification accuracy for each participant. These images were then entered into a second-level one-sample *t*-test to identify voxels that showed significantly higher than chance level classification accuracy (0.50), using the AFNI program 3dttest++. To correct for multiple comparisons, the output group-level statistical map was thresholded using a false discovery rate of *q*(FDR) < 0.05.

## RESULTS

### GROUP-LEVEL GLM

**FIGURE 2:**
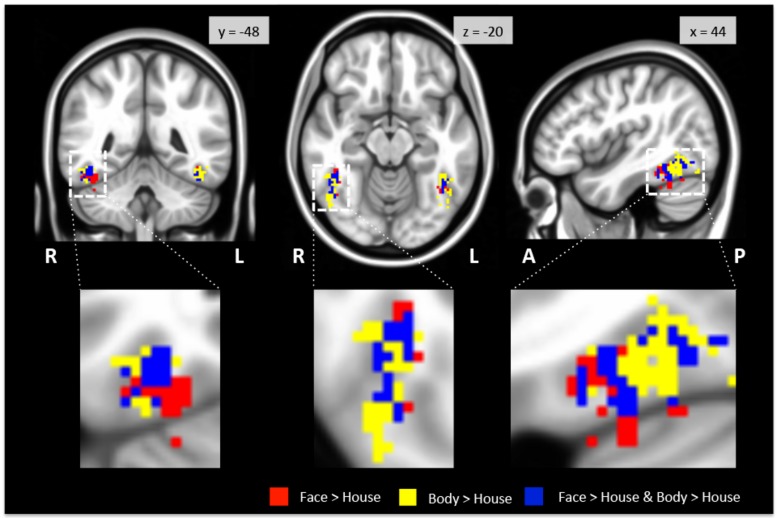
**Regions activated in group-level GLM analysis within the TOFC ROI.** Faces (red) and bodies (yellow) evoked substantially overlapping activations when compared to houses (see **Table 2** for the overall volumes of activations). The blue overlay indicates the overlap between the face > house and body > house contrast maps. The images are centered on the peak activation of the face > house contrast, and activations in the right hemisphere are magnified. R: right, L: left, A: anterior, P: posterior.

As expected, we found bilateral activity within the TOFC ROI for both the face > house and body > house contrasts (peak coordinates in **Table 1**). **Figure 2** displays regions within the TOFC ROI that were significantly activated in the group-level face > house (red) and body > house (yellow) contrasts. The overlap of the face > house and body > house activation maps is also shown (blue).

**Table 1:** MNI coordinates (mm) of group-level peak activations within the TOFC ROI.

	Hemisphere	*X*	*Y*	*Z*	*Z*-score
Face > house	R	44	-48	-20	3.55
	L	-44	-50	-20	4.04
Body > house	R	50	-64	-14	4.35
	L	-44	-50	-20	3.92
Body > face	R	46	-62	-8	4.03
	R	24	-62	-6	3.55
	L	-22	-62	-14	4.25
	L	-46	-70	-16	3.46

*No activations were found for the face > body contrast.*

**Table 2** summarizes the average volume of activation in the face > house and body > house contrast maps and the overlap. In the right hemisphere, the overall volume of activated voxels was 1808 mm^3^ in the face > house contrast and 2760 mm^3^ in the body > house contrast. The overlap of the two contrasts was 976 mm^3^ (54% of the face > house activation). In the left hemisphere, the face > house contrast yielded 1008 mm^3^ of activation, while the body > house activation yielded 2176 mm^3^ with 696 mm^3^ of overlap (69% of the face > house activation). The face > house and body > house contrasts showed peak activity in the same location in the left hemisphere, and the proportion of the overlap was bigger than in the right hemisphere.

**Table 2:** Overall volume of activated voxels from group-level contrasts.

	RH	LH
Face > house	1808 mm^3^	1008 mm^3^
Body > house	2760 mm^3^	2176 mm^3^
Overlap of contrasts (face > house and body > house)	976 mm^3^	696 mm^3^

*Note: RH = right hemisphere, LH = left hemisphere.*

**FIGURE 3:**
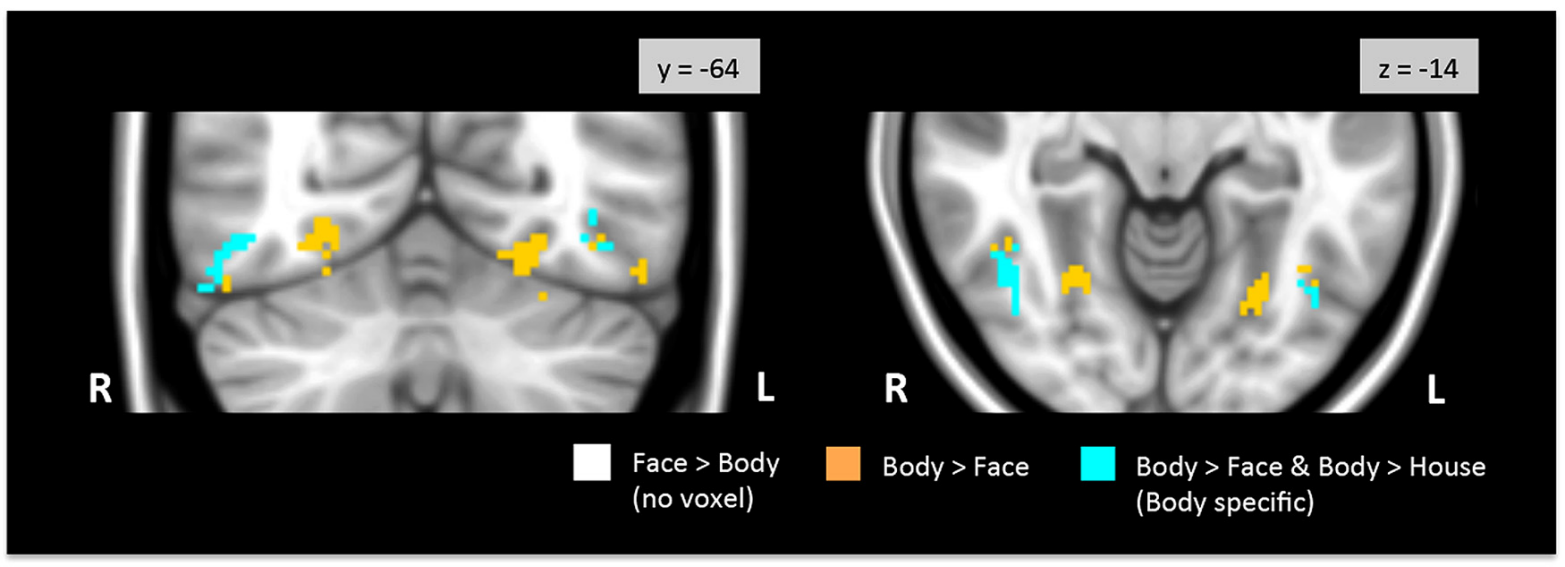
**Direct face vs. body contrasts within the TOFC ROI.** No significantly activated voxels were found in the face > body contrast. Medial and lateral activations were observed in the body > face contrast (orange and cyan). Lateral activations were also found in the body > house contrast, indicating that bodies evoked greater activation in the lateral regions (cyan) than both faces and houses.

**Figure 3** shows regions in the TOFC ROI that were significantly activated in the group-level face > body and body > face contrasts. In the face > body contrast, no significantly activated voxels were found in the fusiform gyrus. However, we found medial and lateral activations in both hemispheres in the body > face contrast (orange and cyan). Peak coordinates for the body > face clusters are also included in **Table 1**. The medial body > face clusters were not observed in the body > house contrast (shown in **Figure 1**). In both hemispheres, the lateral body > face clusters were lateral and posterior to the body > house clusters.

No voxels in the overlap between the face > house and body > house contrast maps were activated when faces and bodies were directly contrasted. Thus, no overlapping voxels showed statistically different activations to faces or bodies.

### GROUP-LEVEL INTERSECTIONS

**FIGURE 4:**
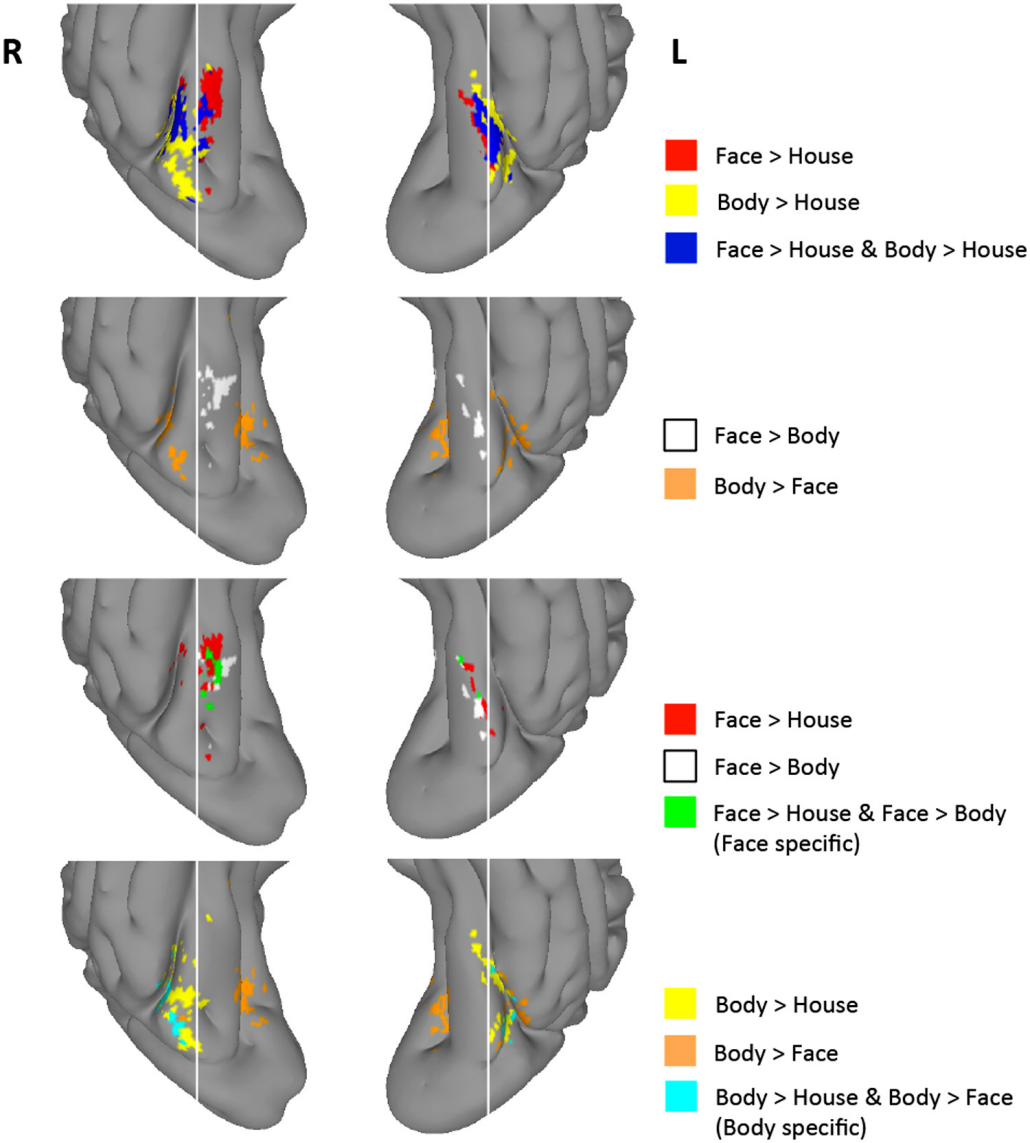
**Group-level uncorrected maps (*Z* > 1.96) within the TOFC ROI.** Regions activated with *Z* > 1.96 in each of the four contrasts and their intersections are indicated in different colors. White vertical lines are overlaid to help compare the spatial distribution of activated regions.

As described above, group-level GLM analyses revealed differential activation maps for faces and bodies in the direct face vs. body contrasts. Despite the strong face-selectivity of the region, we did not find significantly greater activity to faces in the face > body contrast. To guard against a Type II error, we explored the uncorrected statistical maps (*Z* > 1.96) for the four contrasts of interest. **Figure 4** presents regions that were activated in the face > house, body > house, face > body, and body > face contrasts at the uncorrected level. We examined intersections between uncorrected contrast maps to explore the spatial distribution of voxels that exhibited preference to faces and/or bodies in the four contrasts. These analyses were restricted to the TOFC ROI.

As shown in **Figure 4**, 8% of the right hemisphere, and 4% of the left hemisphere voxels from the face > house contrast showed a *Z* > 1.96 for the face > body contrast. These “face specific” voxels (green) appeared on the medial part of the face > house cluster in both hemispheres. 31% of the right hemisphere, and 23% of the left hemisphere voxels from the body > house contrast showed a *Z* > 1.96 for the body > face contrast. These “body specific” voxels (cyan) were found lateral to the body > house clusters.

We also examined whether voxels within the overlap between the face > house and body > house contrasts showed differential activation to faces and bodies in these uncorrected data. We found that some voxels within the overlap exhibited greater activity to faces or bodies (i.e., face > body > house or body > face > house), but the majority (>80%) of the overlapping voxels did not show mean activation difference between faces and bodies (“exclusive overlap”). The size of overlap with no difference between faces and bodies was 52% (right) and 67% (left) of the face > house activations.

### SPATIAL DISTRIBUTIONS OF FACE- AND BODY-EVOKED ACTIVITY

**FIGURE 5:**
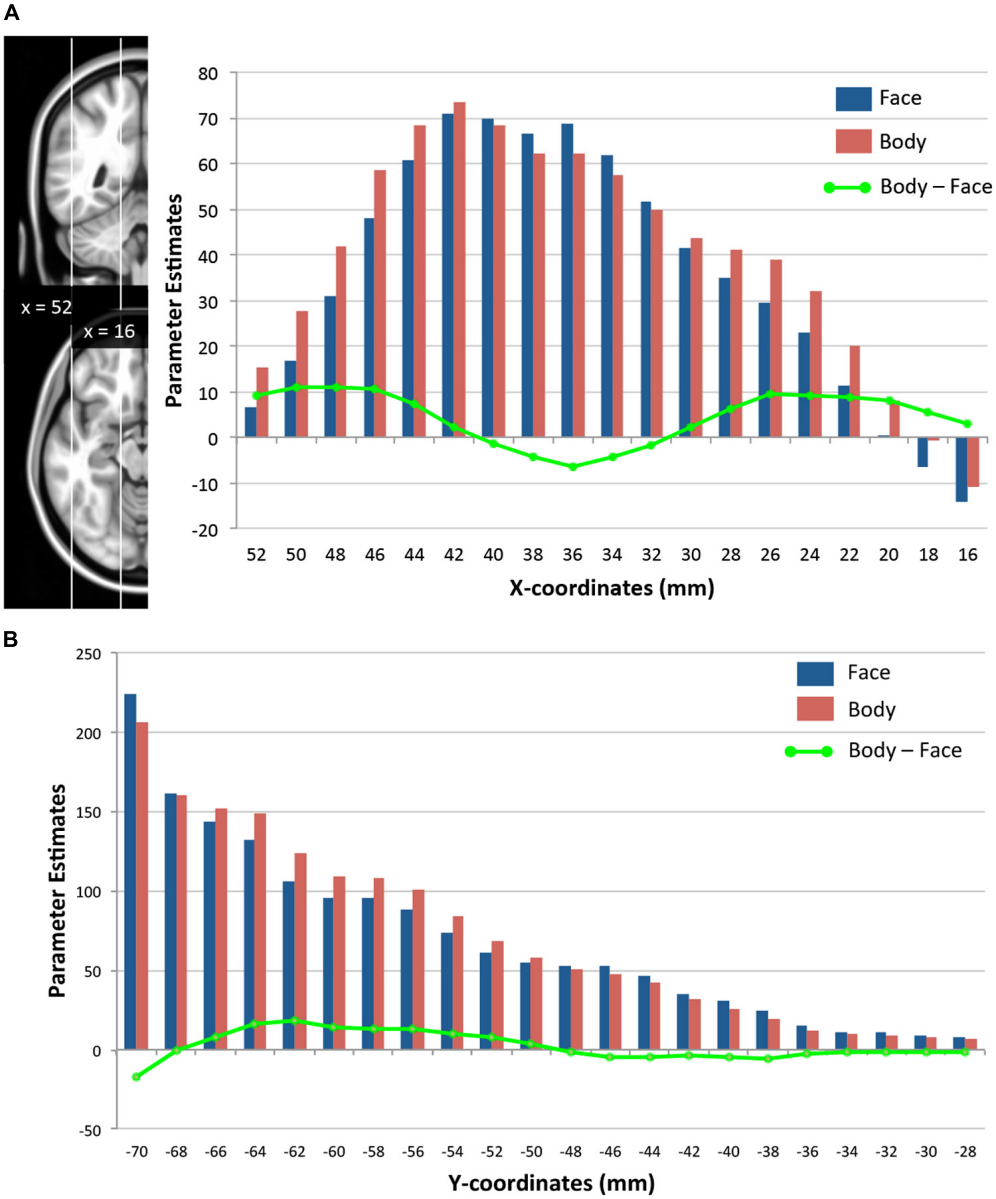
**Spatial distributions of face- and body-evoked activity. (A)** Magnitude of face- and body-evoked activity (relative to baseline) along the lateral–medial axis (x-axis) within the TOFC ROI at x-coordinates (MNI) from 52 mm (lateral) to 16 mm (medial). **(B)** Magnitude of face- and body-evoked activity along the posterior–anterior axis (y-axis) from –70 mm (posterior) to –28 mm (anterior). The green line indicates the difference between faces and bodies at each x- or y-coordinate.

We further examined how the magnitude of face- and body-evoked activity (relative to baseline) varies along the lateral–medial axis (x-axis) and the posterior–anterior axis within the TOFC ROI. As **Figure 5A** shows, face activity was numerically greater than body activity only within the mid-fusiform gyrus (x-coordinates from 32 to 40 mm), and body activity became greater than face activity in areas medial and lateral to the mid-fusiform gyrus – consistent with the GLM results reported above. However, two-tailed one-sample *t*-tests performed on the mean activities at each x-coordinate revealed that the magnitudes of face and body activations were not significantly different. **Figure 5B** shows how face and body activations change along a posterior–anterior axis (y-axis). Because we were interested in the lateral regions where face- and body-evoked activations overlapped, we included only the lateral half of the ROI (from *x* = 36 to 52 mm). Both face and body activity increased toward the posterior, but there was no difference between the two categories. The subtle difference in the magnitude of face- and body-evoked activations is consistent with the GLM results reported above.

### SUBJECT-SPECIFIC RESULTS

Some have argued that face- and body-selective regions in VOTC should be examined in each subject’s anatomical space because anatomical differences between subjects are obscured when coregistering individuals’ data into the template brain for group-level analyses (e.g., Peelen et al., 2006; Weiner and Grill-Spector, 2012). To address this issue, we performed the same analyses in each individual subjects’ anatomical brain space.

As in group-level analyses, we generated uncorrected statistical maps in subject space for the contrasts of interest (i.e., face > house, body > house, face > body, body > face) and obtained the intersections of the maps.

In subject-based analyses, we focused on the proportion of overlap between the face > house and body > house contrasts and the intersection of the face > house and face > body contrasts, in order to compare the size of the overlap relative to the entire face > house activation in each subject. We also calculated the proportion of the face > body voxels among the face > house voxels (“face specific”) in each subject.

On average, 41% of voxels in the right hemisphere and 34% of voxels in the left hemisphere that showed greater activation to faces compared to houses overlapped with the body > house map. Nineteen subjects (out of 21) showed face > body and body > face clusters within the fusiform ROI in both hemispheres. 34% (right hemisphere) and 36% (left hemisphere) of the face > house voxels were also included in the face > body map. Thus, we found substantial overlap between the face > house and body > house maps and a small intersection of the face > house and face > body maps in subject-based analyses, similar to what we have observed in group-level contrast maps.

**FIGURE 6:**
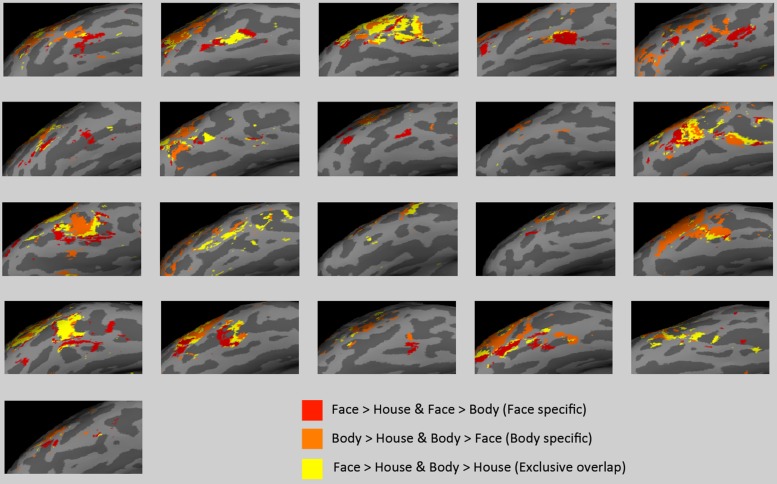
**Activations in subject space.** “Face specific” (red) and “body specific” (orange) regions for each of the 21 subjects are displayed, along with “exclusive overlap” (yellow) regions where face > house and body > house but faces are not different from bodies.

There was large individual variability in the volumes of activation and proportion of the overlap. **Figure 6** presents individual subjects’ functional data on the surface of the lateral VOTC. The “face specific” (face > body and face > house) voxels were displayed in red, the “body specific” (body > face and body > house) in orange, and the “exclusive overlap” (the overlap with no difference between faces and bodies) in yellow.

### MULTI-VOXEL PATTERN ANALYSIS ON THE OVERLAP

We performed MVPA to examine if the voxels that show no activation differences to faces and bodies in univariate GLM analyses could nonetheless discriminate between faces and bodies based on their spatial pattern of activity. Voxels were selected based on the group-level uncorrected statistical maps. 172 voxels in the right hemisphere and 121 voxels in the left hemisphere were selected independently of contiguity.

On average, we found high classification performance (faces vs. bodies vs. houses) in both hemispheres: 58.5% in the right hemisphere and 58.2% in the left hemisphere. A one-sample *t*-test confirmed that these group mean accuracies were significantly above chance level of 33.3% (*p* < 10^-4^). Houses were classified as accurately as faces and bodies within the voxels. As presented in **Table 3**, the confusion matrix showed no preferences. That is, there was no more misclassification between faces and bodies, as between faces and houses, or between bodies and houses.

**Table 3:** Confusion matrices from three-way classification of face, body, and house blocks.

Actual category	Classified category		
	Face	Body	House
**RH (172 voxels)**
Face	0.59	0.20	0.21
Body	0.21	0.58	0.21
House	0.20	0.21	0.59
**LH (121 voxels)**
Face	0.55	0.21	0.24
Body	0.22	0.65	0.13
House	0.28	0.17	0.55

*Values represent average fraction (across participants) of face, body, and house blocks each classified as face, body, or house blocks. An ideal confusion matrix (i.e., perfect classification) would have values of 1 on the diagonal (grayed entries) and values of 0 on the off-diagonal entries (errors). RH, right hemisphere; LH, left hemisphere.*

Thus, the activation patterns within the overlapping voxels were discriminable among our three categories even though these voxels did not show a mean activation difference between faces and bodies.

### MULTIVARIATE SEARCHLIGHT ANALYSIS

**FIGURE 7:**
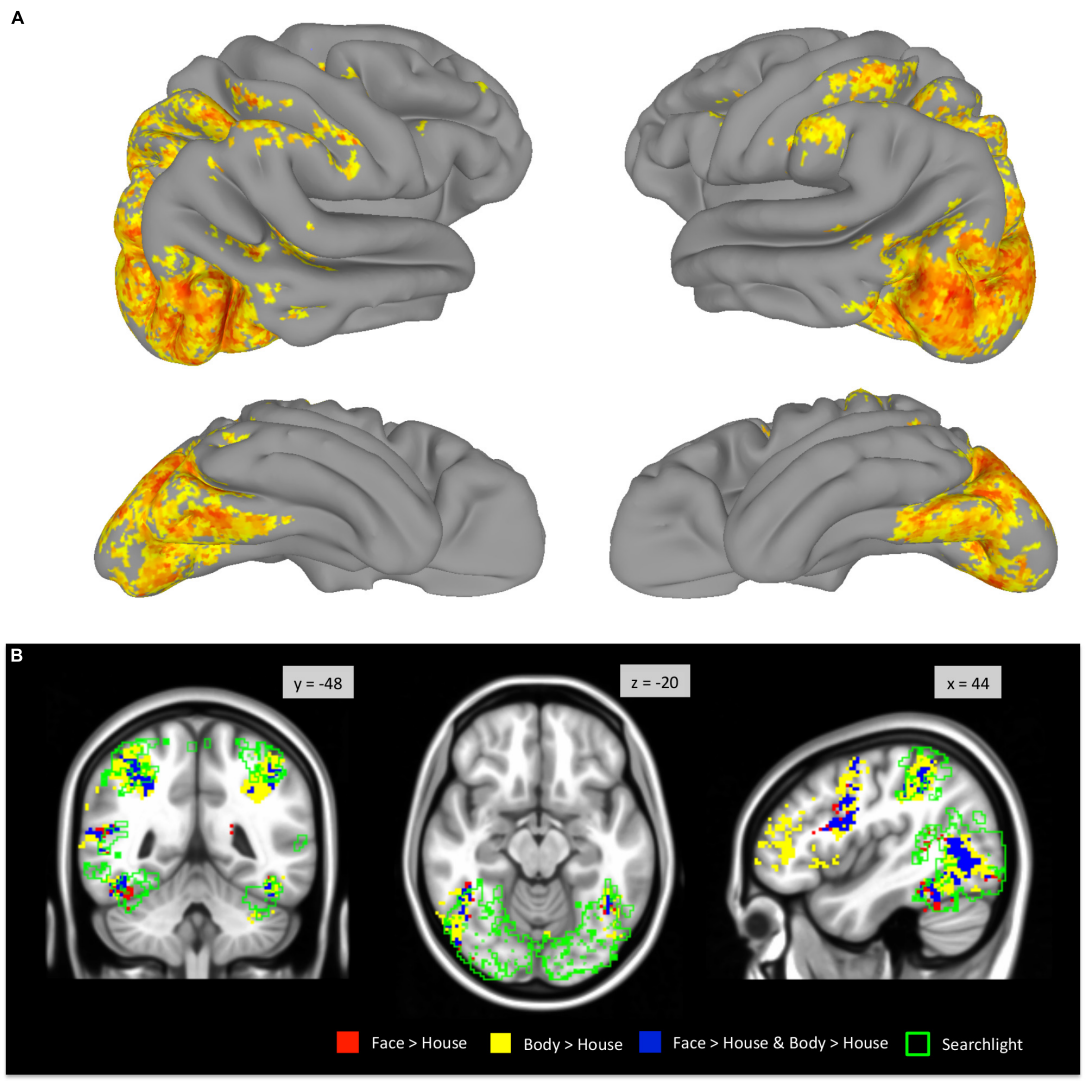
**Whole-brain searchlight analysis. (A)** Areas that survived FDR correction are displayed on the surface of the standard brain. **(B)** Voxels that survived FDR correction are outlined in green and overlaid on the group-level GLM contrast maps (derived from **Figure 2**).

A whole-brain searchlight analysis demonstrated that faces and bodies are highly accurately decoded by local patterns of activity within VOTC and within the occipital lobe (**Figure 7**). Extensive regions where classification accuracies above chance level were obtained included bilateral VOTC that extended to the occipital regions, and the supramarginal gyri. Within the VOTC, faces and bodies were discriminable above chance in regions beyond the boundaries of those that selectively responded to faces and/or bodies compared to houses. **Figure 7B** overlays the group-level searchlight results upon the regions that showed mean activation differences between faces and bodies. Most of the voxels in the TOFC ROI that strongly respond to faces and/or bodies also contained local pattern differences between the two categories, even without mean activation differences.

## DISCUSSION

There is agreement in the literature that the perception of both faces and bodies activates regions of the VOTC, principally within the fusiform gyrus. At issue is the degree to which these activations overlap or are anatomically distinct, and thus provide evidence for a highly modular or more distributed neural architecture. The overlap in activation in the VOTC evoked by the perception of faces and bodies has been observed in prior studies (e.g., Morris et al., 2006, 2008; Spiridon et al., 2006), as has the finding that some voxels are more strongly activated by one or the other category (e.g., Schwarzlose et al., 2005; Weiner and Grill-Spector, 2010). Our results indicate more overlap than has been reported in prior studies. In the right hemisphere, we observed that 54% of the face > house activation overlapped with the body > house contrast, while other groups have reported less than 30% on average (Schwarzlose et al., 2005; Weiner and Grill-Spector, 2010). Furthermore, following correction for multiple comparisons, we found no voxels in which faces evoke more activity than bodies in group level analyses, and relatively few voxels showing such differences in uncorrected statistical comparisons.

The shapes of the spatial distributions of activation for faces and bodies in the medial-lateral extent of the fusiform gyrus were similar. However, despite having similarly located spatial peaks (i.e., where the beta values were at maximum), the spatial distribution of activation for bodies was somewhat kurtotic in appearance relative to faces – i.e., somewhat flatter in the middle and with more activity in the medial and lateral tails. The group-level GLM revealed a significant body > face contrast in both the medial and lateral tails of the distribution. The voxels in the medial aspect of the distribution tail did not differentiate bodies from houses, and thus recalls Morris et al. (2006) who argued that a medial fusiform activation by bodies was not a domain specific process. However, the voxels in the lateral tail of the spatial distributions *did* reveal voxels that were more strongly activated by bodies than by faces *and* by houses. It may be, then, that it is these voxels that compose the fusiform body area, although the bulk of these voxels were within or lateral to the inferior occipital sulcus and thus located in the inferior temporal gyrus. It is notable, however, that the activation evoked by bodies in this lateral region is less than half of that observed in the mid-fusiform gyrus where faces and houses evoke nearly equivalent activation. That is, the VOTC area in which significant differences between faces and bodies were obtained in GLM was at the lateral periphery of *both* the face and body fusiform activations.

If the existence of voxels where faces and bodies evoke significantly different levels of activation is evidence for a discrete neural instantiation of a modular processing stream, what then, does overlap represent? Does the overlap represent a hemodynamic or vascular smearing or other spatial blurring of otherwise discrete neural representations? Is it an artifact of combining data across subjects? Or is this evidence for a functional convergence of face and body activations? Hemodynamic smearing or other blurring would seem more likely to occur in a region between two spatially distinct peaks. However, as we have seen, the medial–lateral peak of the face and body activations were roughly the same in the mid-fusiform, and the area showing the strongest body > (face and house) response is lateral to both peaks. While it is very likely that combining across subjects contributed to some of the observed overlap, our individual subject analysis revealed substantial overlap of face and body activations, with overlap as high as 80% of activated voxels in the right fusiform gyrus of one individual.

The issue of functional convergence is less easily addressed. Using MVPA, we observed that the pattern of activation within the region of overlap, where no mean activation differences between faces and bodies were present, still contained sufficient information to discriminate faces from bodies. The confusion matrices for three-way classification indicated that the classifier did not simply distinguish faces from non-face stimuli, or bodies from non-body stimuli. Good classification accuracies in regions of overlap were found at both the group-level and individual subject analysis. These results are compatible with the idea that faces and bodies have an intermixed or patchy representation at a finer scale within a larger face and body sensitive area (Pinsk et al., 2009; Weiner and Grill-Spector, 2010) and support the suggestion from earlier studies (e.g., Grill-Spector et al., 2006; Çukur et al., 2013) that the VOTC, and the FFA in particular, has a more heterogeneous organization than previously appreciated.

Discriminable activation patterns for faces and bodies have been previously reported. For example, Weiner and Grill-Spector (2010) used a winner-take-all classifier to identify faces or body parts among six object categories (faces, body parts, houses, flowers, guitars, and cars; chance level 17%). Within the voxels in lateral ventral temporal cortex that showed selective response to faces or body parts (i.e., the union of face-selective and limb-selective voxels), accuracies of 97% for faces and 94% for body parts were found. However, these accuracies are difficult to directly compare to the present study as different classifier methods and different ROIs were used. We have applied a more restrictive criterion for voxel selection and included a relatively small number of examples (i.e., the number of blocks for each stimulus type), which might have reduced classification performance (O’Toole et al., 2007; Etzel et al., 2009). Our results show that, even in the voxels that fall within overlapping activations, faces and bodies were discriminated above chance in the patterns of activity. This finding suggests that the representations of faces and bodies converge in some regions of VOTC, but remain nevertheless discriminable.

This interpretation is consistent with a recent intracranial EEG study from our laboratory (Engell and McCarthy, 2014) in which ERPs recorded from subdural electrodes showed strong selectivity to different corporeal stimuli (faces, isolated eyes, and headless bodies) compared to a control category (flowers) from sites along the fusiform gyrus and surrounding cortex. However, most sites that were selective to one type of corporeal stimulus were also sensitive to the other types – that is, there were few sites that responded exclusively to one type of corporeal stimulus – and only one of 1536 electrode sites examined in 12 subjects showed a specific response to bodies compared to faces and isolated eyes. Engell and McCarthy did find, however, instances in which the different corporeal stimuli evoked a difference in the spatial distribution of voltage over closely spaced adjacent electrodes – suggesting that faces, bodies, and eyes engaged a different configuration of current sources and sinks, despite activating the same electrodes. Engell and McCarthy concluded that this was evidence consistent with a lumpy or patchy representation of corporeal stimuli that may be evident at a finer spatial resolution than that offered by fMRI.

However, other studies have suggested that regions of the fusiform gyrus identified as face-selective in standard localizer tasks respond strongly to such stimuli as dynamic point-light displays of human ambulation (Engell and McCarthy, 2013) and the purposeful, or causal, movements of machines that are otherwise devoid of human surface characteristics (Shultz and McCarthy, 2012). This suggests that at least some of the regions of VOTC identified as face-selective by standard localizer tasks integrate information about social or intentional agents – and may as a consequence show task-related variation in patterns of activation in addition to stimulus-related variation. Based upon intracranial ERP studies, we previously suggested that there may be a temporal course whereby areas initially responding to an exemplar of a specific stimulus category (perhaps reflected by the initial stimulus-driven N200 ERP recorded directly from the fusiform gyrus) followed by a period where the initial representation is modified by other stimulus and task factors and perhaps reflected in the subsequent gamma activity at the same electrode sites (Puce et al., 1999; Engell and McCarthy, 2010).

Our paper has focused upon the fusiform gyrus and on functional regions defined in the extensive literature on high-level visual perception as the FFA and FBA using typical methods for identifying these regions. However, we also used a whole-brain searchlight MVPA approach to explore for other regions that could discriminate faces from bodies at above chance levels. Extensive regions of the VOTC beyond the operationally defined FFA and FBA could significantly discriminate faces from bodies, as could the posterior STS, the supramarginal gyrus and intraparietal sulcus. Faces and bodies are visually very different, and so perhaps it is not surprising that many visual regions of the brain can discriminate these stimuli. Of course this same concern applies to the discriminability between faces and bodies observed in the overlap region of our operationally defined FFA and FBA. Our results demonstrate differential pattern information specific to faces and bodies, but the current study does not address what specific information within faces or bodies is represented in the patterns of activity. Given the enhanced sensitivity of multi-voxel patterns to more specific information compared to univariate analyses, future research may be able to investigate whether more specific features of a face or body image, rather than generic categorical information, can also be decoded by activation patterns in VOTC.

To conclude, the current study investigated the similarities and distinctiveness of face- and body-evoked activations in VOTC, specifically in the fusiform gyrus. The results support that regions in VOTC maintain functional specificity for faces and bodies, even though the two categories were not differentiated in mean activation levels within those regions. This study also exemplifies the use of univariate and multivariate analyses in investigating similar but disparate activations of a local brain region.

## Conflict of Interest Statement

The authors declare that the research was conducted in the absence of any commercial or financial relationships that could be construed as a potential conflict of interest.
